# Supplementary figures and images for: MrTADFinder: A network modularity based approach to identify topologically associating domains in multiple resolutions
Source: PLoS Comput Biol. 2017 Jul 24;13(7):e1005647. doi: 10.1371/journal.pcbi.1005647 (PMC5546724; doi:10.1371/journal.pcbi.1005647)

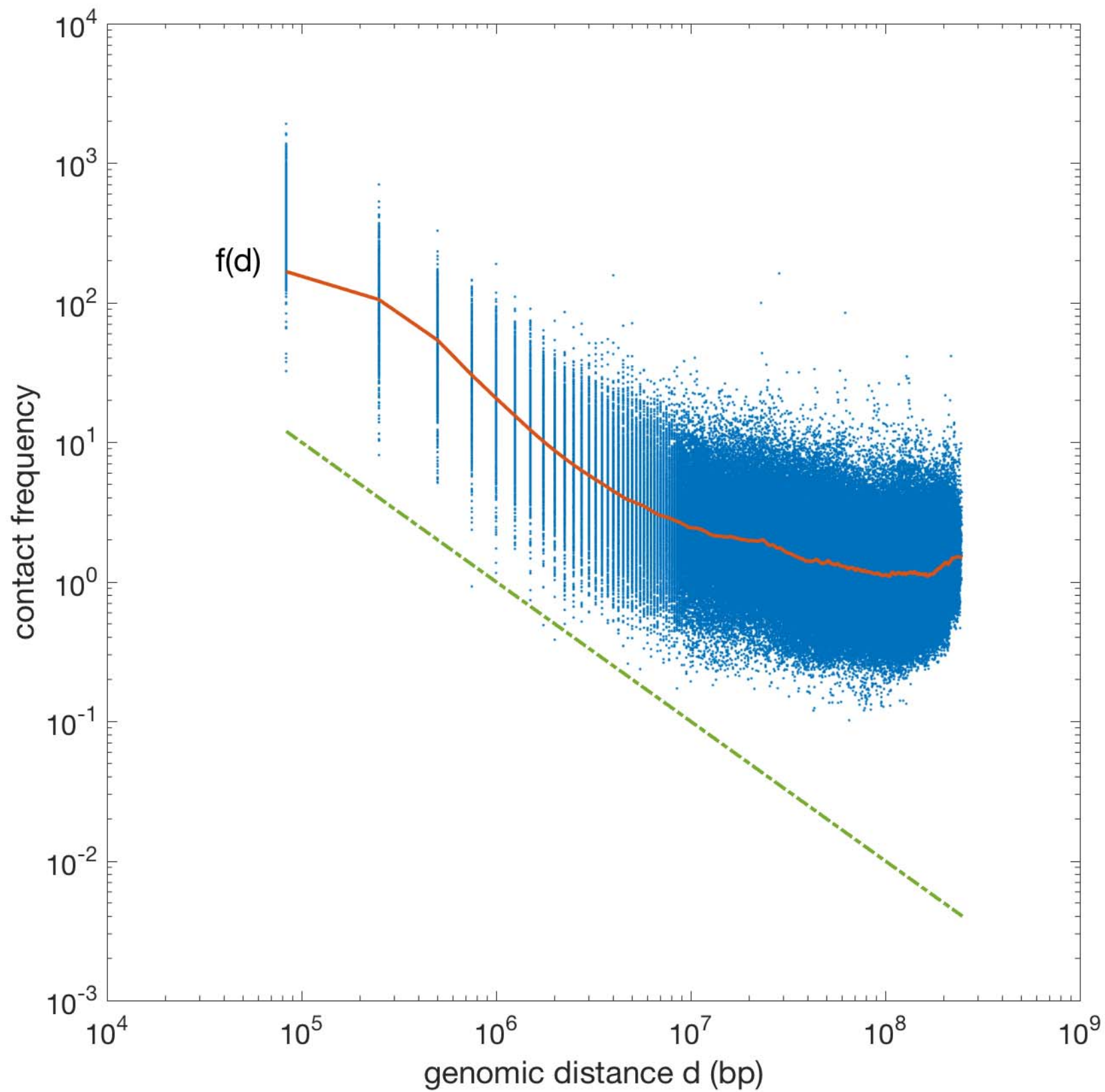

Supplement: S1 Fig — The analysis was performed using the contact map of the chromosome 1 of MCF7, binned in 250kb sized bins. The red line f(d) is the average contact frequency as a function of distance d obtained by smoothing. The green line shows a power-law function d−1. (PDF) [file pcbi.1005647.s001.pdf]

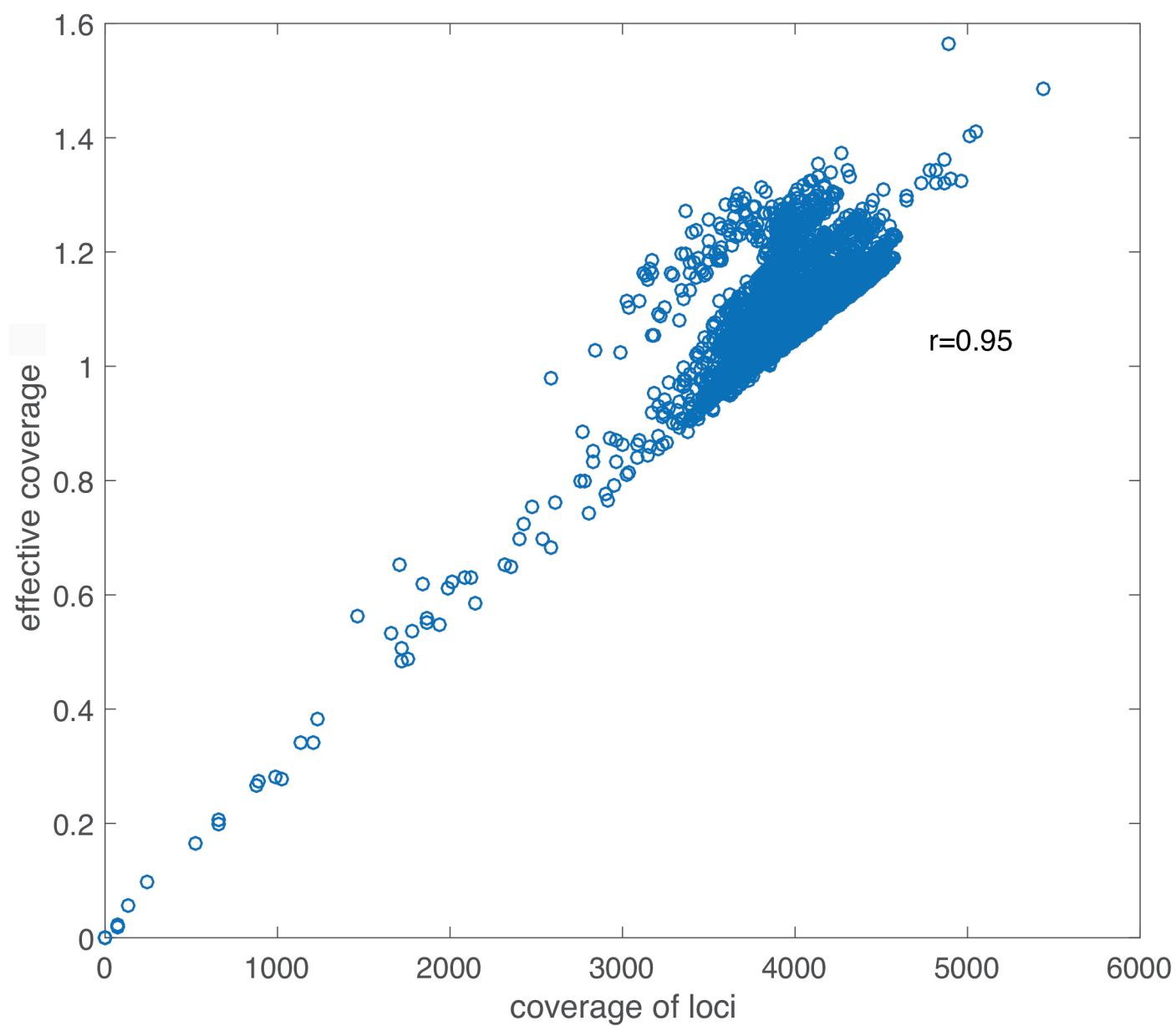

Supplement: S2 Fig — (PDF) [file pcbi.1005647.s002.pdf]

hESC: chr 10

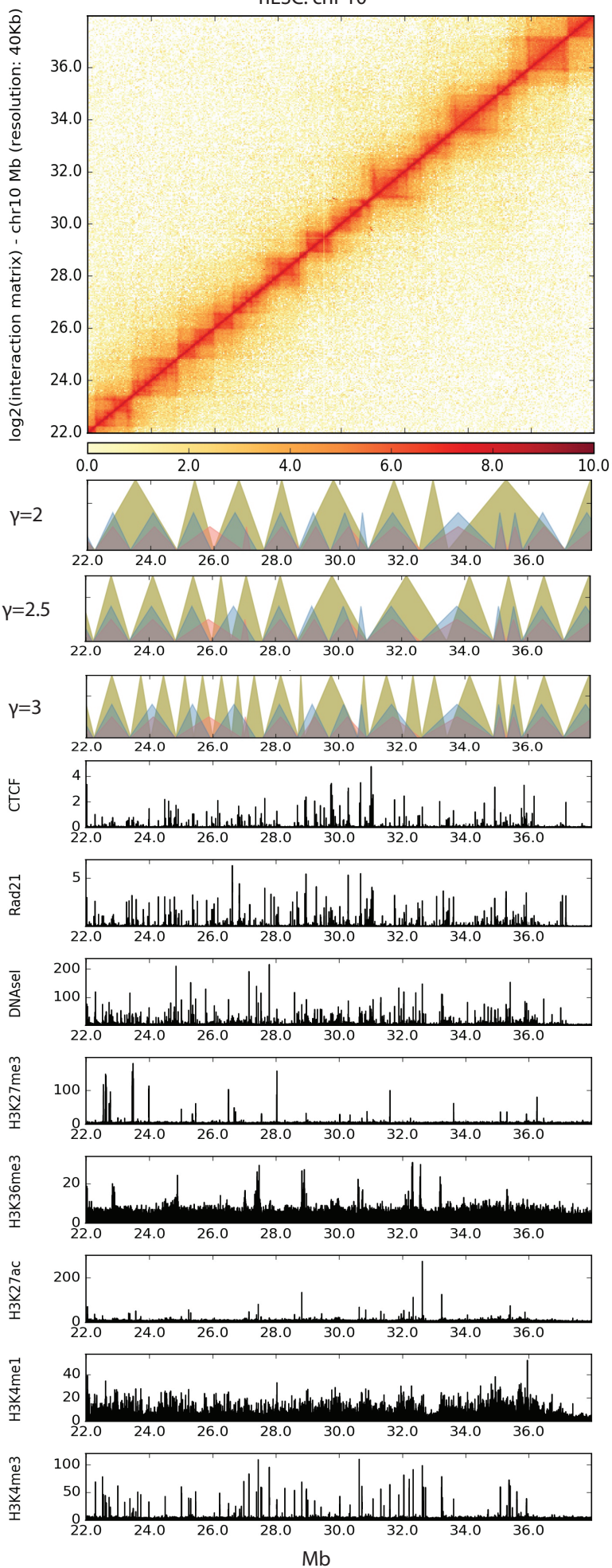

Supplement: S3 Fig — (PDF) [file pcbi.1005647.s003.pdf]

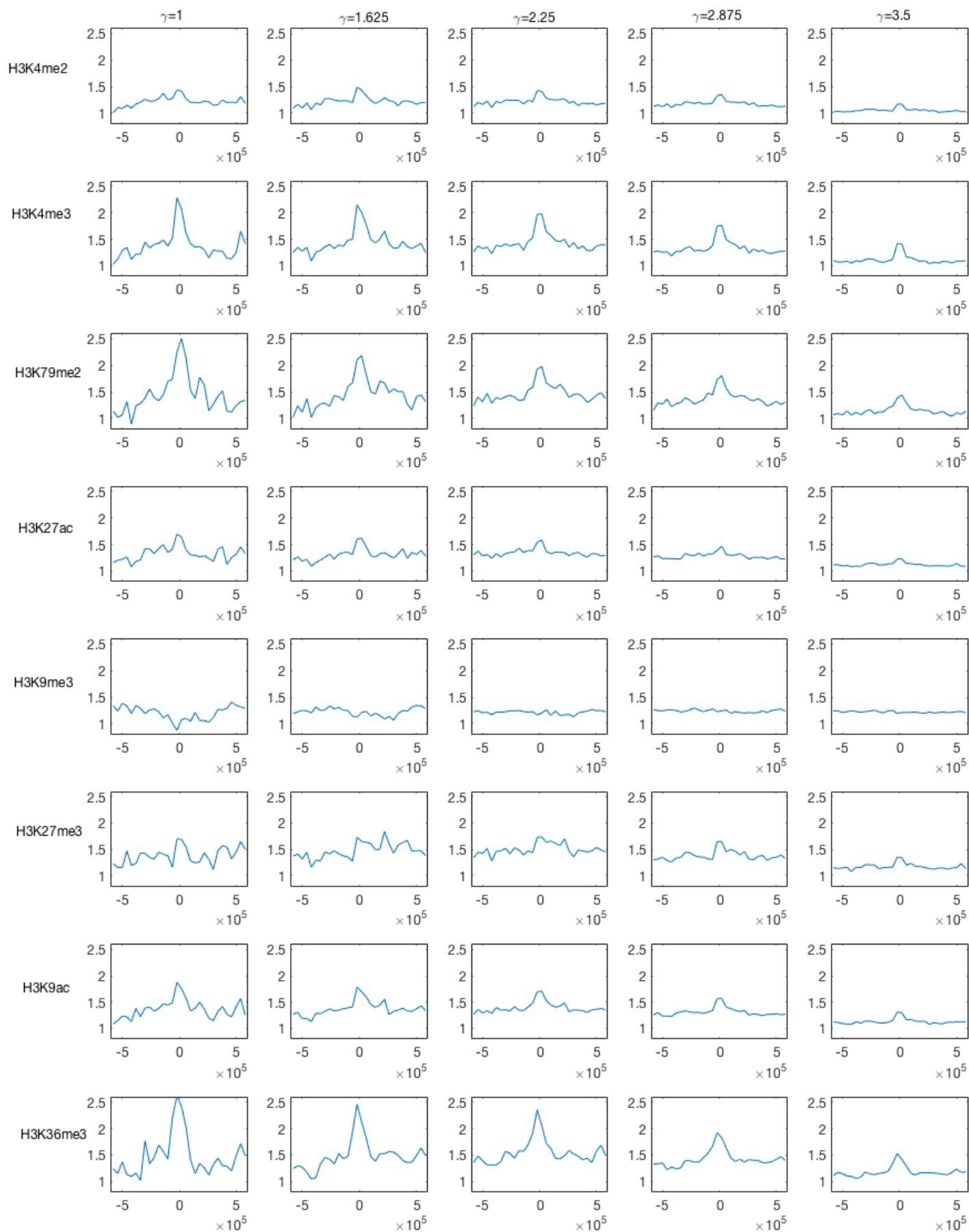

Supplement: S4 Fig — (PDF) [file pcbi.1005647.s004.pdf]

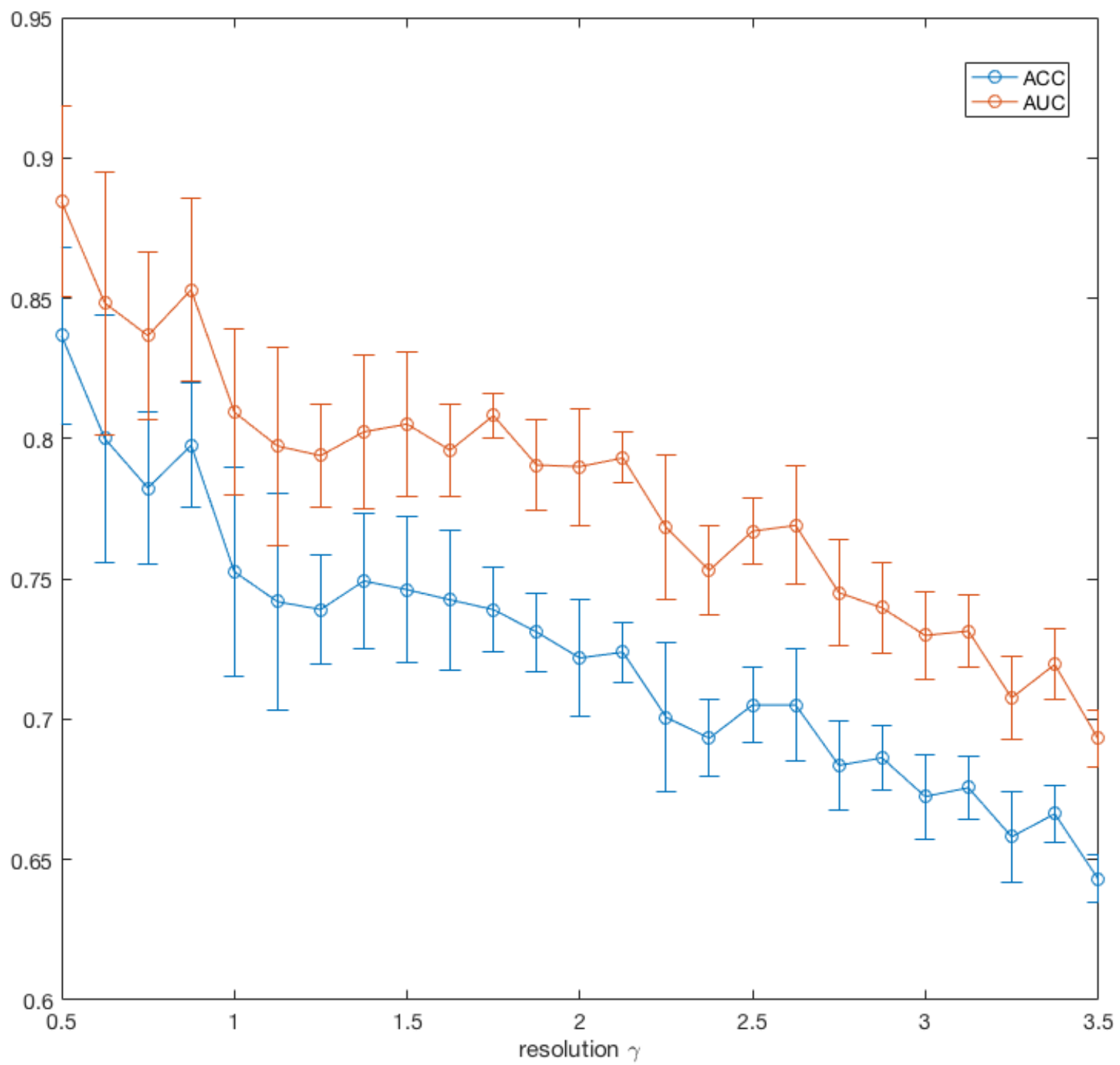

Supplement: S5 Fig — For each resolution, a logistic regression model based on transcription factors binding signals was trained to classify the TAD boundaries versus a set of random boundaries. The error bars were estimated by repeating the analysis using an ensemble of random boundaries. The performance (AUC and ACC) decreases as the resolution increases. (PDF) [file pcbi.1005647.s005.pdf]

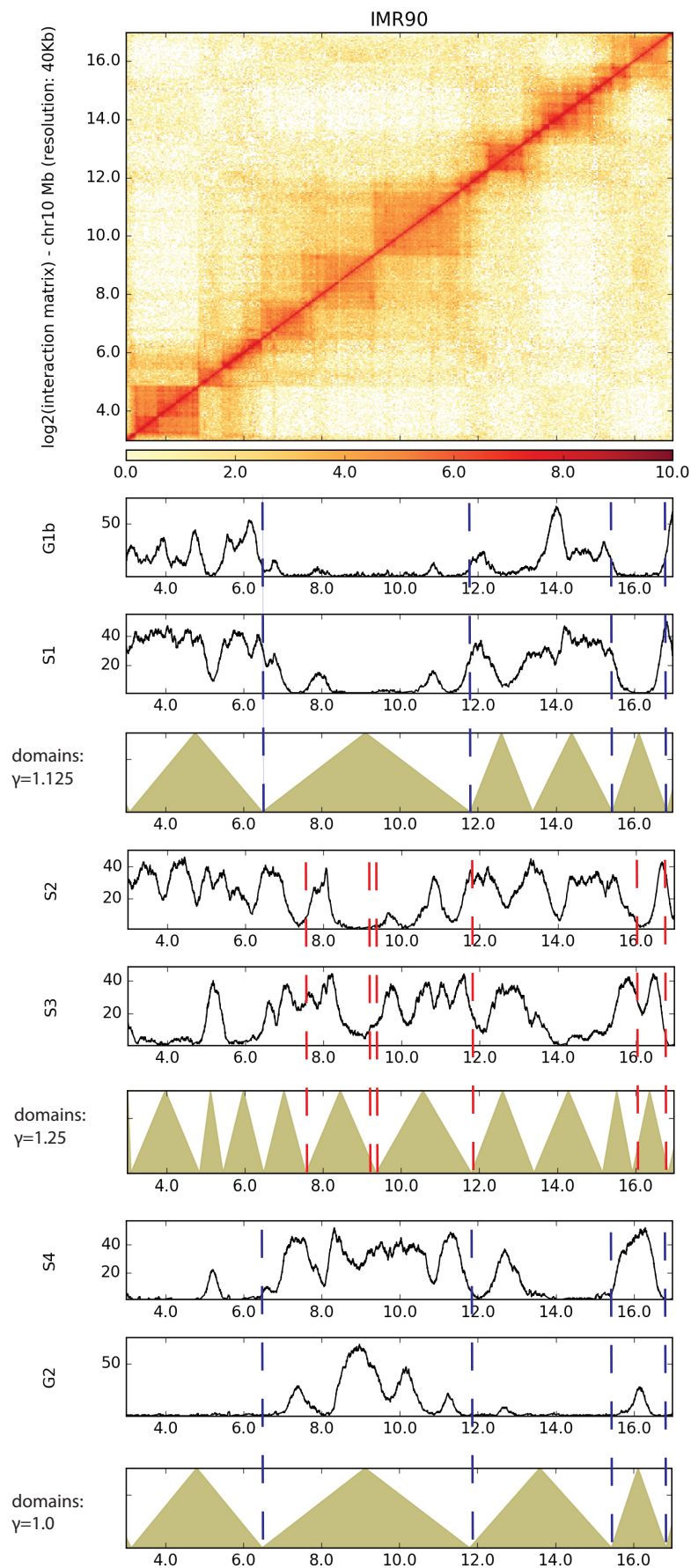

Supplement: S6 Fig — TADs are identified for IMR90 using different resolutions. Signals of Repli-seq data in various stages of a cell cycle and a part of the contact map of the chromosome 10 are displayed. The TADs match visually well with the replication timing signals. The middle TAD identified in γ = 1 does not replicate at S1, its sub-units identified in γ = 1.25 replicate in S2 and S3.as shown by the peaks in the Repli-seq signal. (PDF) [file pcbi.1005647.s006.pdf]

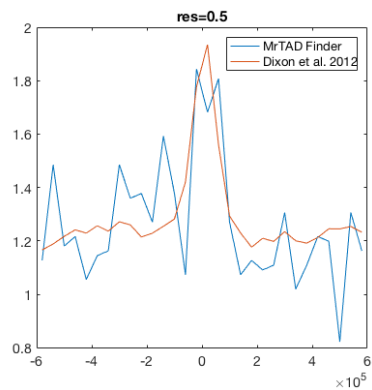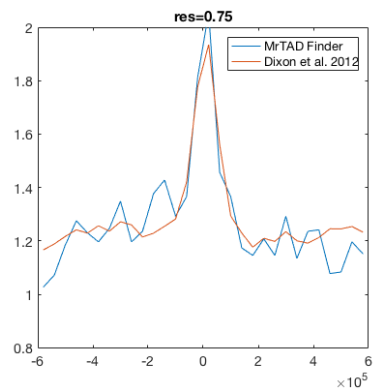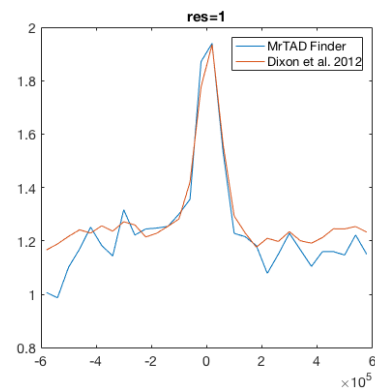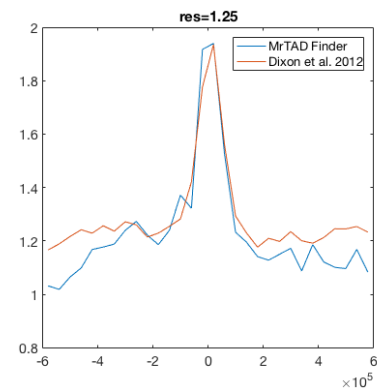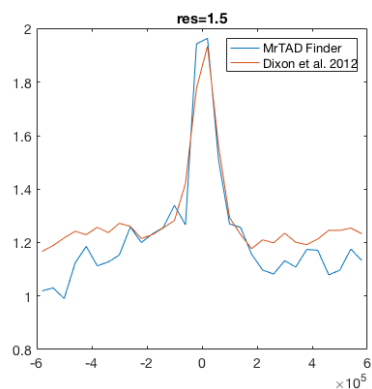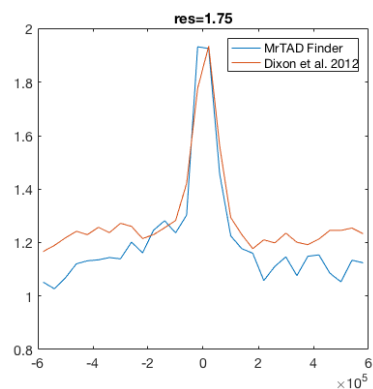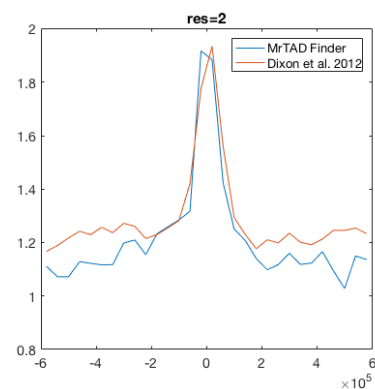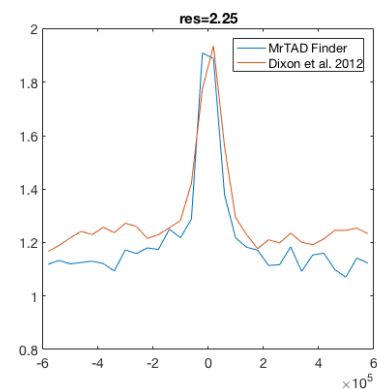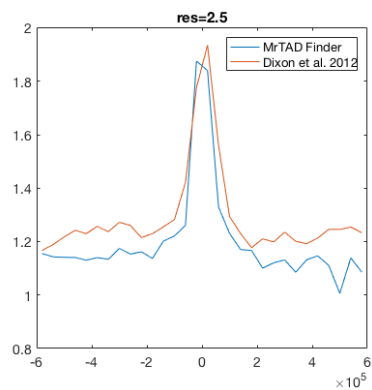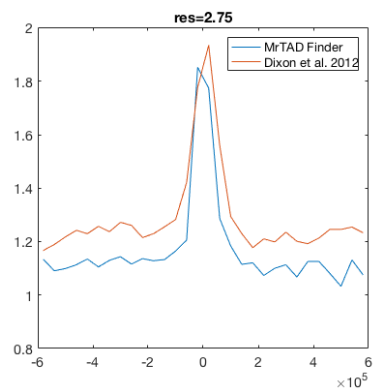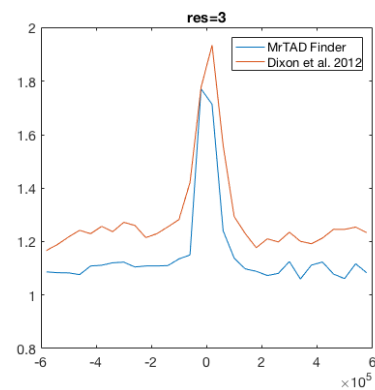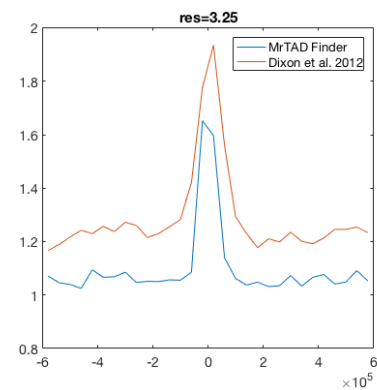

Supplement: S7 Fig — The red line shows the same analysis using TADs reported in [8]. This figure is an extension of Fig 8. (PDF) [file pcbi.1005647.s007.pdf]

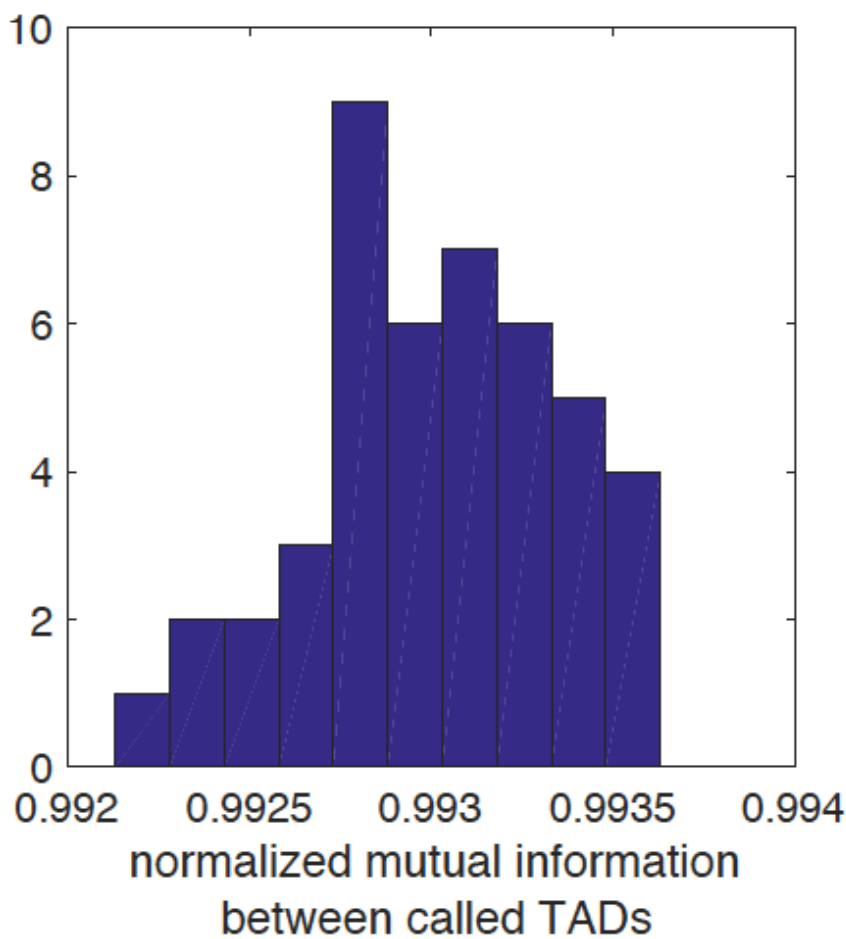

Supplement: S8 Fig — Histogram for pairs of independently called TADs. Using the default parameters (10 trials of the modified Louvain algorithm and a cut-off of 0.9), the normalized mutual information between two sets of called domains agrees extremely well (nMI = 0.99). (PDF) [file pcbi.1005647.s008.pdf]

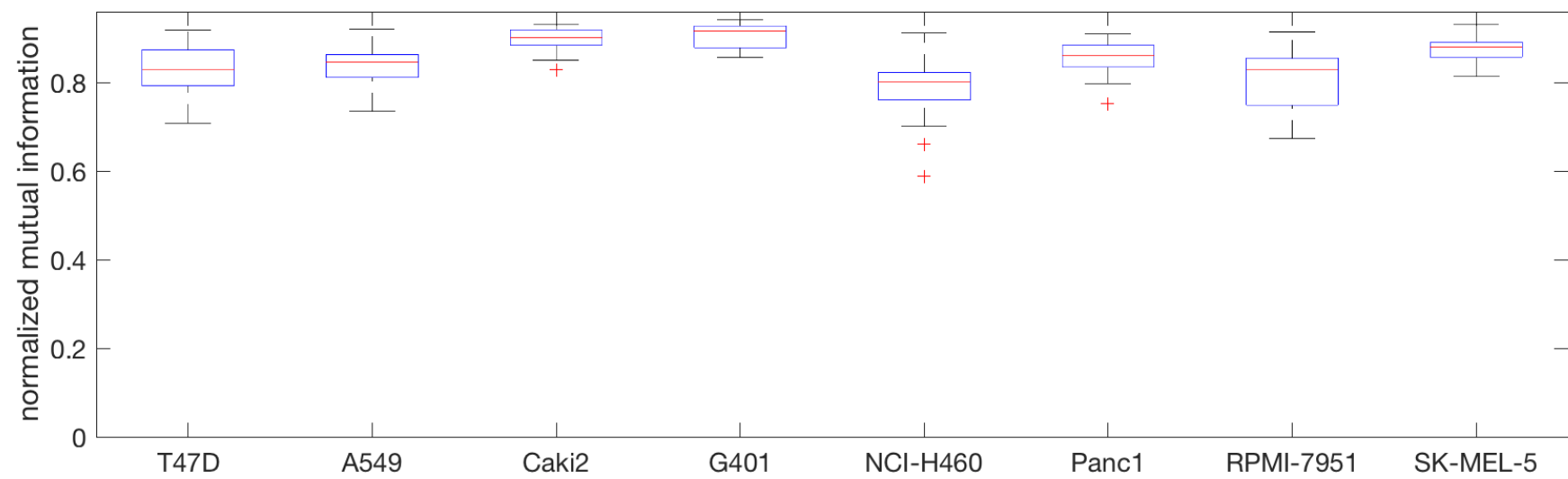

Supplement: S9 Fig — For each cell line, TADs were called separately in each replicate for all chromosomes. The boxplot shows the distribution of the normalized mutual information for 23 chromosomes in different cell lines. (PDF) [file pcbi.1005647.s009.pdf]

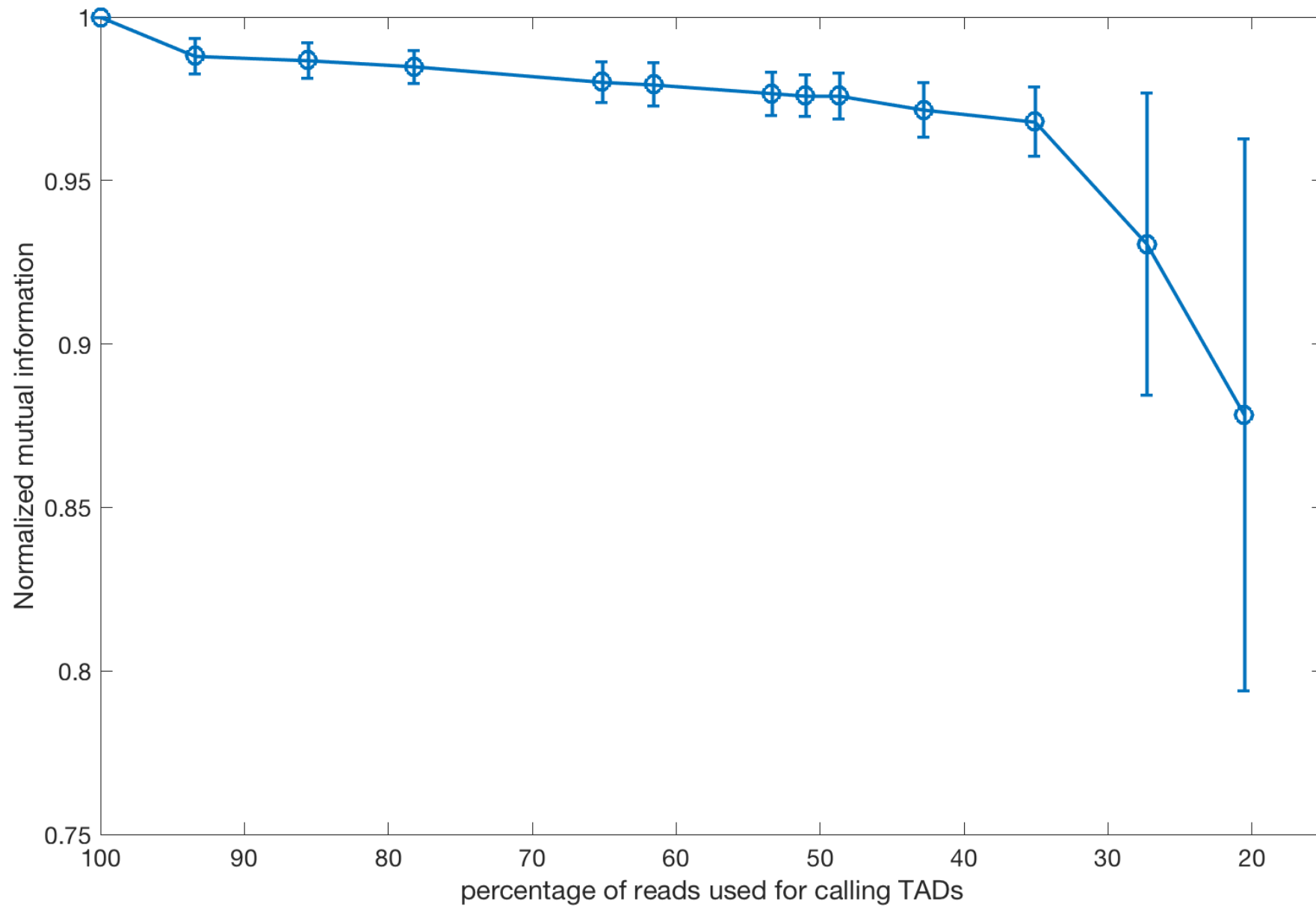

Supplement: S10 Fig — An original set of TADs was identified from contact maps constructed for 2.4 billion reads. Subsequent sets of TADs were called by reducing the number of reads. The discrepancy with the original set quantified by normalized mutual information. For each comparison, the average normalized mutual information of different pairs of chromosomes is plotted in the y-axis, whereas the errorbar shows the corresponding standard deviation. Despite a certain level of discrepancy, the resultant TADs agree well. (PDF) [file pcbi.1005647.s010.pdf]

$$M_{ij} = \text{optQ}(i(i+1)(i+2)...j)$$

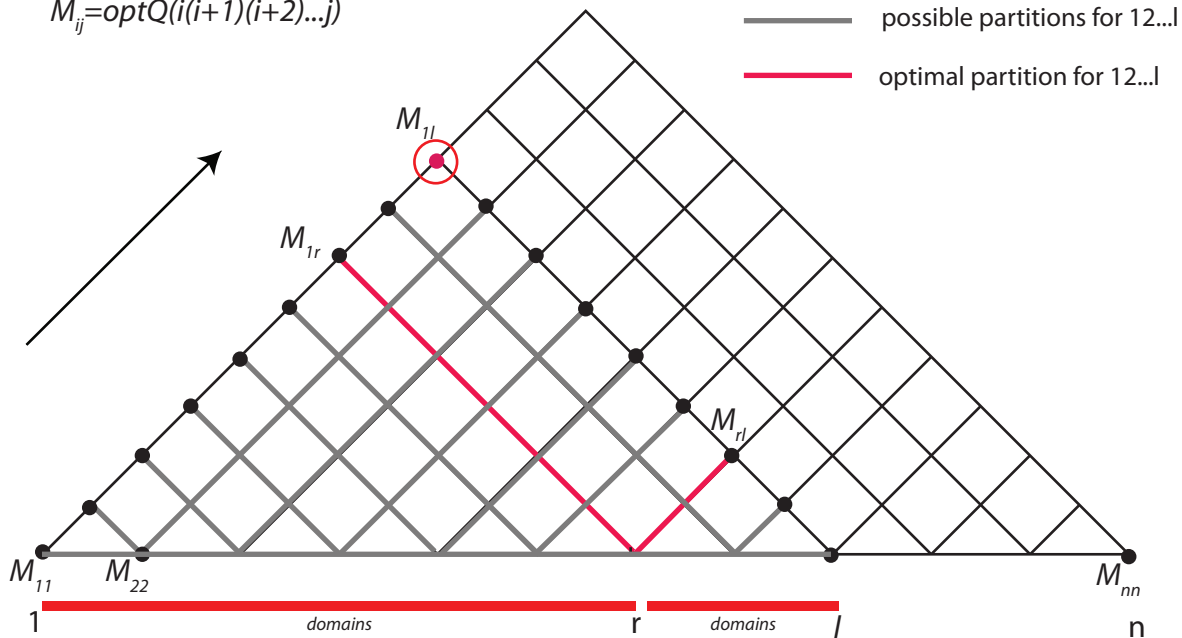

Supplement: S11 Fig — The optimal value of Q for a chromosome segment running from i to j is stored in Mij. The values of all elements in M can be enumerated using dynamic programming, starting from fragments of length 1 where Mii = Qii. There are different ways to divide a fragment of length l (gray lines). Suppose the optimal way is marked by the red line, then M1l = M1r + Mrl. (PDF) [file pcbi.1005647.s011.pdf]
